# Role of interferon-gamma release assay for screening and monitoring of latent tuberculosis infection in kidney transplant recipients

**DOI:** 10.1186/s12879-024-09990-x

**Published:** 2024-10-07

**Authors:** Jackrapong Bruminhent, Tanadon Treekajonsak, Surasak Kantachuvesiri, Chavachol Setthaudom, Warawut Sukkasem, Theerasuk Kawamatawong

**Affiliations:** 1 Division of Infectious Diseases, Department of Medicine, and Ramathibodi Excellence Center for Transplantation, Faculty of Medicine Ramathibodi Hospital, Mahidol University, 270 Rama VI Road, Ratchathewi, Bangkok, 10400 Thailand; 2 Ramathibodi Excellence Center for Organ Transplantation, Faculty of Medicine Ramathibodi Hospital, Mahidol University, Bangkok, Thailand; 3 Department of Medicine, Faculty of Medicine Ramathibodi Hospital, Mahidol University, Bangkok, Thailand; 4 Division of Nephrology, Department of Medicine, Faculty of Medicine Ramathibodi Hospital, Mahidol University, Bangkok, Thailand; 5 Immunology Laboratory, Department of Pathology, Faculty of Medicine Ramathibodi Hospital, Mahidol University, Bangkok, Thailand; 6 Department of Diagnostic and Therapeutic Radiology, Faculty of Medicine Ramathibodi Hospital, Mahidol University, Bangkok, Thailand; 7 Division of Pulmonary and Critical Care Medicine, Department of Medicine, Faculty of Medicine Ramathibodi Hospital, Mahidol University, Bangkok, Thailand

**Keywords:** ELISpot, Immunocompromised, Cell-mediated immunity, IGRA, TB disease

## Abstract

**Background:**

The reactivation of tuberculosis (TB) among kidney transplant (KT) recipients in an endemic area is of general concern. However, the epidemiology of latent TB infection (LTBI) status and its dynamic change responses have not been explored.

**Methods:**

Between September 2020 and August 2021, a prospective study was conducted to investigate the status of LTBI in KT recipients who received a 9-month isoniazid universal prophylaxis. This status was measured using the interferon-gamma release assay (IGRA) with T-SPOT.*TB* before transplant, as well as at one month and nine months post-transplant.

**Results:**

Ninety-one KT recipients had a mean (SD) age of 45 (11) years, and 41% were female. Sixty-eight (75%) patients received a deceased donor allograft, and eighty-six (91%) patients received induction immunosuppressive therapy. The IGRA results were positive, borderline, negative, and indeterminate in 14 (15.4%), 6 (6.6%), 64 (70.3%), and 7 (7.8%) patients, respectively. Among 84 evaluable patients, 20 (23.8%) KT recipients were defined as having LTBI. Older age was significantly associated with LTBI (OR 1.06 [95% CI 1.01–1.12], *p* = 0.03). Among the 77 KT recipients who completed monitoring, 55 had negative IGRA results. Three (5.4%) KT recipients had conversion post-transplant. One of them developed pulmonary TB at 1 week after the transplant. Among the 13 patients with positive results, 8 (61.5%) remained positive, 1 (7.7%) had an indeterminate result at 1-month post-transplant and subsequently tested positive at 9 months post-transplant, and 4 (30.8%) experienced reversion to negative results throughout the study.

**Conclusions:**

In a high TB-endemic area, one-quarter of KT recipients were reported to have LTBI, and the dynamic change of IGRA response in KT recipients is plausible post-transplant.

**Supplementary Information:**

The online version contains supplementary material available at 10.1186/s12879-024-09990-x.

## Introduction

Tuberculosis (TB) is one the most common infections with a high prevalence in developing countries. Immunocompromised individuals, such as solid organ transplant (SOT) recipients, are more vulnerable than the general population to TB from TB reactivation and new TB infection. This devastating infection can result in substantial morbidity and mortality due to their impaired immune function from immunosuppressive agents [1]. The incidence of active TB disease among kidney transplant (KT) recipients is much higher than in the general population and TB is also responsible for the loss of the transplanted kidney in approximately one-third of cases [2]. In developed countries, TB incidence in KT recipients is 0.5−6.5%, but it is 3.1−15.4% in endemic countries [3]. The reported prevalence of post-transplant TB is 3.1 to 15% in Asia, and about 45–60% of TB occurs in the first year after transplantation [1]. Most of the TB cases in KT recipients are due to the reactivation of latent TB infection (LTBI) [2]. According to Guidelines from the infectious disease community of practice of the American Society of Transplantation, several preventive treatments should be considered for all transplant patients who have evidence of LTBI without a history of adequate treatment [4]. Despite this recommendation, screening is not routinely performed owing to economic constraints or lack of accessibility to such tests in resource-constrained countries. Some experts recommend universal isoniazid (INH) prophylaxis for candidates in highly endemic areas during the first year after transplantation, with maximum immunosuppression [4]. TB prevention strategies after KT that have been utilized vary across Thailand institutions such as universal INH prophylaxis or close monitoring for TB presentation after transplant without determining LTBI status. The role of universal INH prophylaxis has to be balanced between the benefit and risk of hepatotoxicity from the chemoprophylactic agent. On the other hand, observing TB symptoms could place patients at risk of severe, disseminated TB while being immunosuppressed. In addition, transmission of TB may occur to others. Furthermore, there is limited information about the true epidemiology of LTBI among KT population residing in endemic area particularly in Thailand. Additionally, the role of dynamic change of TB-specific immunity among KT recipients has not been explored. The primary objective of this study was to investigate the incidence and risk factors for LTBI in KT recipients. The secondary objective was to monitor the dynamic change of IGRA results after KT. The inclusion criteria included all adult recipients who underwent KT during study period.

## Materials and methods

### Study design

Between September 2020 and August 2021, a prospective study to investigate LTBI status among KT recipients was conducted at Faculty of Medicine Ramathibodi Hospital, Mahidol University, Bangkok, Thailand. All participants received universal INH prophylaxis for 9 months regardless of their IGRA status after transplant. We did not perform directly observed therapy; however, medication adherence was encouraged and assessed at each visit.

The participants were excluded if they were diagnosed with active TB at the time of enrollment, terminated surgery due to any cause, or if their peripheral blood mononuclear cell (PBMC) sample was inadequate (defined as less than 2.5 × 10^5^) for testing.

They were screened for LTBI by interferon-gamma release assay (IGRA) using the T-SPOT.*TB* before transplant (M0) and monitored a dynamic change at one month (M1) and nine months (M9) post-transplant. LTBI was defined as a positive IGRA result without TB symptoms and without an abnormal chest radiograph (pleural thickening, fibrotic scarring, or pulmonary nodules (calcified or non-calcified) [5, 6]. Conversion was defined as those with a negative IGRA result before transplant which converted to positive result after transplant. Reversion were those with a positive result which turned negative thereafter.

### Interferon-gamma release assay (IGRA) using the T-SPOT.*TB* test

The T-SPOT.*TB* test (Oxford Immunotec, Oxford, England) was conducted by adding 2.5 × 10^5^ PBMC to four wells of a 96-well plate pre-coated with anti-interferon-gamma antibody. Phytohaemagglutinin (PHA) was added to the positive control well, the negative control well, TB antigen (TBAg) with the early secreted antigenic target 6-kDa protein (ESAT-6) or culture filtrate protein 10 (CFP-10) peptide pools were added to two antigen wells. The plates were incubated at 37 °C with 5% CO_2_ for 16–20 h. Post incubation, the wells were washed with phosphate-buffered saline, developed using an anti-interferon-gamma antibody conjugate, and a substrate was added to detect secreted interferon-gamma. Spot-forming cells were counted with an automated ELISPOT reader, and low-level counts in unstimulated wells were subtracted from the test well results. The maximum number of spot-forming units (SFU) between two antigens provided SFU/2.5 × 10^5^ PBMC. Results were categorized as positive (≥ 8 spots), negative (≤ 4 spots), borderline (5–7 spots), or indeterminate (positive control < 20 spots and antigen wells ≤ 4 spots, or nil control > 10 spots). The larger value between ESAT-6 and CFP-10 was defined as the TBAg/PHA ratio [7, 8]. Those with borderline results were considered as positive for comparative analysis. The test showed 100% specificity in low-risk populations and 98.8% sensitivity in immunocompromised patients [9]. 

#### Data collection

We collected clinical demographic data (age, gender, body mass index [BMI], etiologies of end-stage kidney disease [ESKD], underlying disease), transplant data (type of transplant, immunosuppressive drug regimen), IGRA results (positive, negative, indeterminate) and LTBI (chest x-ray, signs and symptoms of TB, history of TB).

### Statistical analysis

The Continuous variables were summarized as mean and standard deviation (SD), and categorical variables were summarized as frequencies and percentages.

Continuous variables were compared by a Student’s *t*-test. Categorical variables were were compared by a χ^2^ test or Fisher’s exact test as appropriated. The clinical characteristic data and outcomes were assessed with descriptive statistics. The incidence of LTBI was summarized as percentages, and risk factors of LTBI were assessed by logistic regression analysis. Values of *p* < 0.05 were considered significant. All Statistical analysis will be performed by Stata statistical software, version 15 (StataCorp, LLC, College Station, TX, USA).

## Results

### Study population

**Fig. 1:**
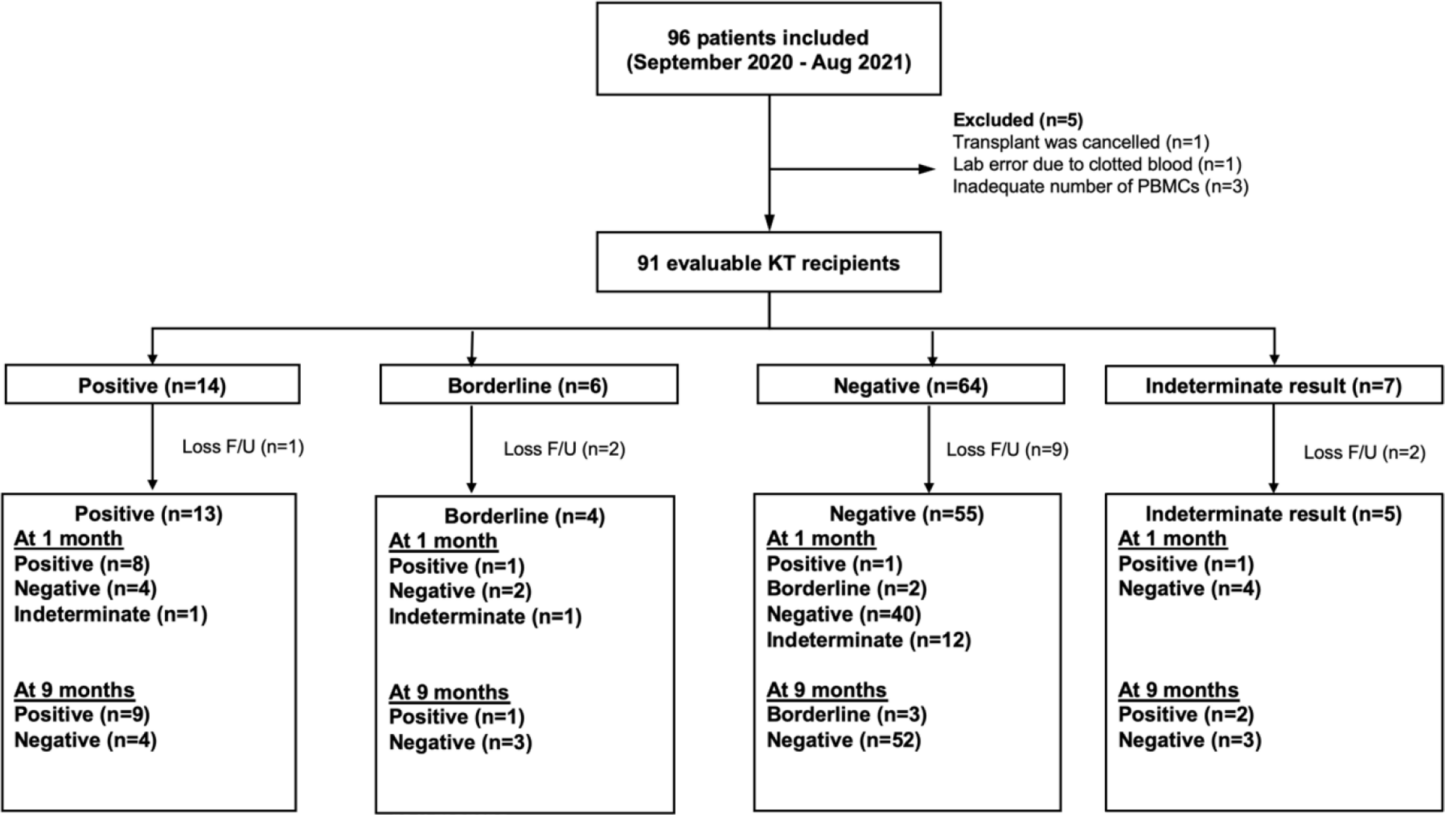
Study flow. **Abbreviations** PMBCs, peripheral blood mononuclear cells

A total of ninety-six patients underwent KT from September 2020 to August 2021 (Fig. 1). Five patients were excluded due to clotted blood (*n* = 1), inadequate number of PBMCs (*n* = 3), and terminated surgery (*n* = 1). The remaining ninety-one evaluable patients were testing using the T-SPOT.*TB* test before KT.

Among 91 KT recipients, the mean (SD) age was 44 [11] years old, and 41% of those were female. The most prevalent comorbidities were hypertension (81%), hyperparathyroidism (31%), and diabetes mellitus (12%). There were two patients who had a prior diagnosis of TB disease. Sixty-eight (75%) patients received a deceased donor allograft and 84 (92%) patients underwent the first transplantation. Eighty-six (91%) patients received induction immunosuppressive therapy. The maintenance immunosuppression therapy included prednisolone (100%), tacrolimus (96%) and mycophenolate mofetil (77%).

### Latent TB infection

Among 91 KT recipients who underwent IGRA testing, the results were positive, borderline, negative and indeterminate in 14 (15.4%), 6 (6.6%), 64(70.3%), and 7(7.8%) patients, respectively (Fig. 1). Among 84 evaluable patients, 20 (23.8%) KT recipients were defined as having LTBI. The characteristics of 84 participants with positive (including borderline results) and negative were compared and shown in Table 1. Older age was significantly associated with LTBI in KT recipients (OR 1.06 [95%CI 1.01–1.12], *p* = 0.03) in logistic regression analysis (Table 2).

**Table 1:** Patients characteristics of KT recipients with and without pre-transplant status LTBI status

Characteristics *n* (%)	Total (*n* = 84)	LTBI (*n* = 20)	No LTBI (*n* = 64)	*P*-value
Age, year (mean ± SD)	44.36 ± 10.65	49.25 ± 9.45	43.17 ± 10.57	0.05
Male	51 (61)	12 (60)	39 (61)	0.69
BMI, kg/m^2^ (mean *±* SD)	22.65 *±* (3.54)	22.54 *±* 3.21	22.72 *±* 3.63	0.94
Comorbidities				0.64
DM	11 (13)	2 (10)	9 (14)	
Hypertension	69 (82)	15 (75)	54 (84)	
Dyslipidemia	4 (5)	2 (10)	2 (3)	
HBV infection	4 (5)	3 (15)	1 (2)	
HCV infection	1 (1)	0 (0)	1 (2)	
Hyperparathyroidism	27 (32)	7 (35)	20 (31)	
Systemic lupus erythematosus	3 (4)	1 (5)	2 (3)	
Coronary artery Disease	3 (7)	1 (5)	2 (3)	
COPD	1 (1)	1 (5)	0 (0)	
Previous TB infection	2 (2)	0(0)	2 (3)	> 0.99
Donor age-year (mean ± SD)	39.64 ± 14.05	41 ± 12.12	39.28 ± 11.76	0.85
Type of allograft				0.29
Deceased donor	64 (76)	17 (85)	47 (73)	
Living-related	20 (24)	3 (15)	17 (27)	
Second KT	6 (7)	2 (10)	4 (6)	0.57
HLA mismatch				0.94
< 3	51 (61)	12 (60)	39 (61)	
≥ 3	33 (39)	8 (40)	25 (39)	
PRA (%)				0.47
10 or less	65 (77)	17 (85)	48 (75)	
11 to 50	9 (11)	1 (5)	8 (13)	
51 or more	10 (12)	2 (10)	8 (13)	
Induction immunosuppression				0.79
IL-2 receptor antagonist	60 (71)	15 (75)	45 (70)	
Anti-thymocyte globulin	19 (23)	3 (15)	16 (25)	
No induction	5 (6)	2 (10)	3 (5)	
Maintenance immunosuppression				0.63
Tacrolimus	80 (95)	18 (90)	62 (97)	
Mycophenolate mofetil	64 (76)	16 (80)	48 (75)	
Prednisolone	84 (100)	20 (100)	64 (100)	
Sirolimus	0 (0)	0 (0)	0 (0)	
Cyclosporin A	5 (6)	3 (15)	2 (3)	
Mycophenolate sodium	19 (23)	3 (15)	16 (25)	
Everolimus	0 (0)	0 (0)	0 (0)	

**Abbreviations** LTBI: Latent tuberculosis Infection; SD: Standard deviation; DM: Diabetes mellitus; HBV: Hepatitis B virus; HCV: Hepatitis C virus; COPD: Chronic obstructive pulmonary disease; TB; Tuberculosis; KT: Kidney transplant; HLA: Human leukocyte antigen; PRA: Panel reactive antibody; IL-2: Interleukin-2

**Table 2:** Risk factors for LTBI in KT recipients by logic regression analysis

Factors	OR	95% CI	*P* value
Age (per 1 year)	1.06	1.01–1.12	0.03
Female sex	0.96	0.33–2.68	0.94
BMI (per 1 kg/m^2^)	0.96	0.84–1.11	0.61
Underlying diabetes	1.29	0.25–6.62	0.76

**Abbreviations** LTBI: Latent tuberculosis infection; OR: Odd ratio; Cl: Confidence interval; BMI: Body mass index; kg/m^2^: Kilogram per square meter

### Dynamic change of IGRA test after transplant

Dynamic changes of IGRA test results after transplant are shown in Fig. 1. Among 55 patients with negative results, 2/55 (3.6%) had borderline results, 12/55 (21.8%) had indeterminate results, 40/55 (72.7%) remained negative, and 1/55 (1.8%) had conversion at 1 month post-transplant. The latter developed pulmonary TB at 1 week post-transplant without report of TB investigation in the donor. The result turned negative after TB treatment at 9 months post-transplant. Those with borderline results did not develop TB; one remained borderline and turned negative after 9 months. For the rest, two had conversion and two had borderline results at 9 months post-transplant without clinical symptoms of TB. The rest remained negative.

Among 4 patients with borderline results, 1/4 (25%) had a positive result, 1/4 (25%) had an indeterminate result, and 2/4 (50%) had negative results at 1-month post-transplant. At 9 months, 1/4 (25%) had a persistently positive result without TB symptoms, and 3/4 (75%) had negative results.

Among 13 patients with positive results, 8/13 (61.5%) remained having positive results, 1/13 (7.7%) had an indeterminate result, and 4/13 (30.8%) had reversion results at 1-month post-transplant. At 9 months, 9 had positive results (including 1 patient with an indeterminate result who converted), and the 4 patients with reversion remained negative.

Among 5 patients with indeterminate results, 1/5 (20%) tested positive and 4/5 (80%) tested negative at 1-month post-transplant. At 9 months post-transplant, the former reverted to negative, and half of the rest converted to positive results without TB symptoms.

**Fig. 2:**
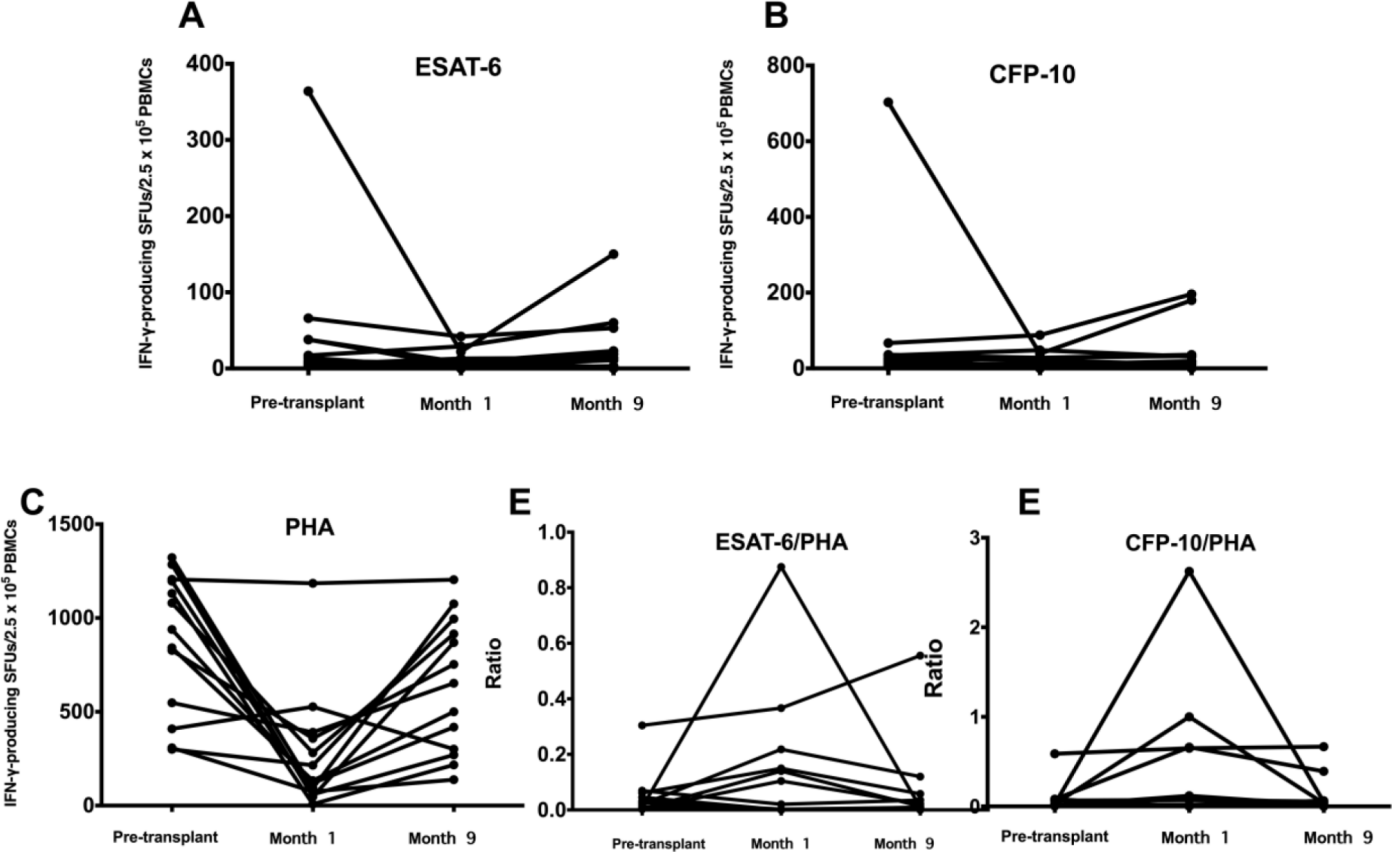
A dynamic plot of of TB antigen-specific T cell responses among those with positive IGRA tests. **Abbreviations** CFP-10, culture filtrate protein-10; ESAT-6, early secreted antigenic target 6-kDa protein; PHA, phytohaemagglutinin; PBMCs, peripheral blood mononuclear cells; TBAg, TB antigen; SFU, spot-forming units

**Fig. 3:**
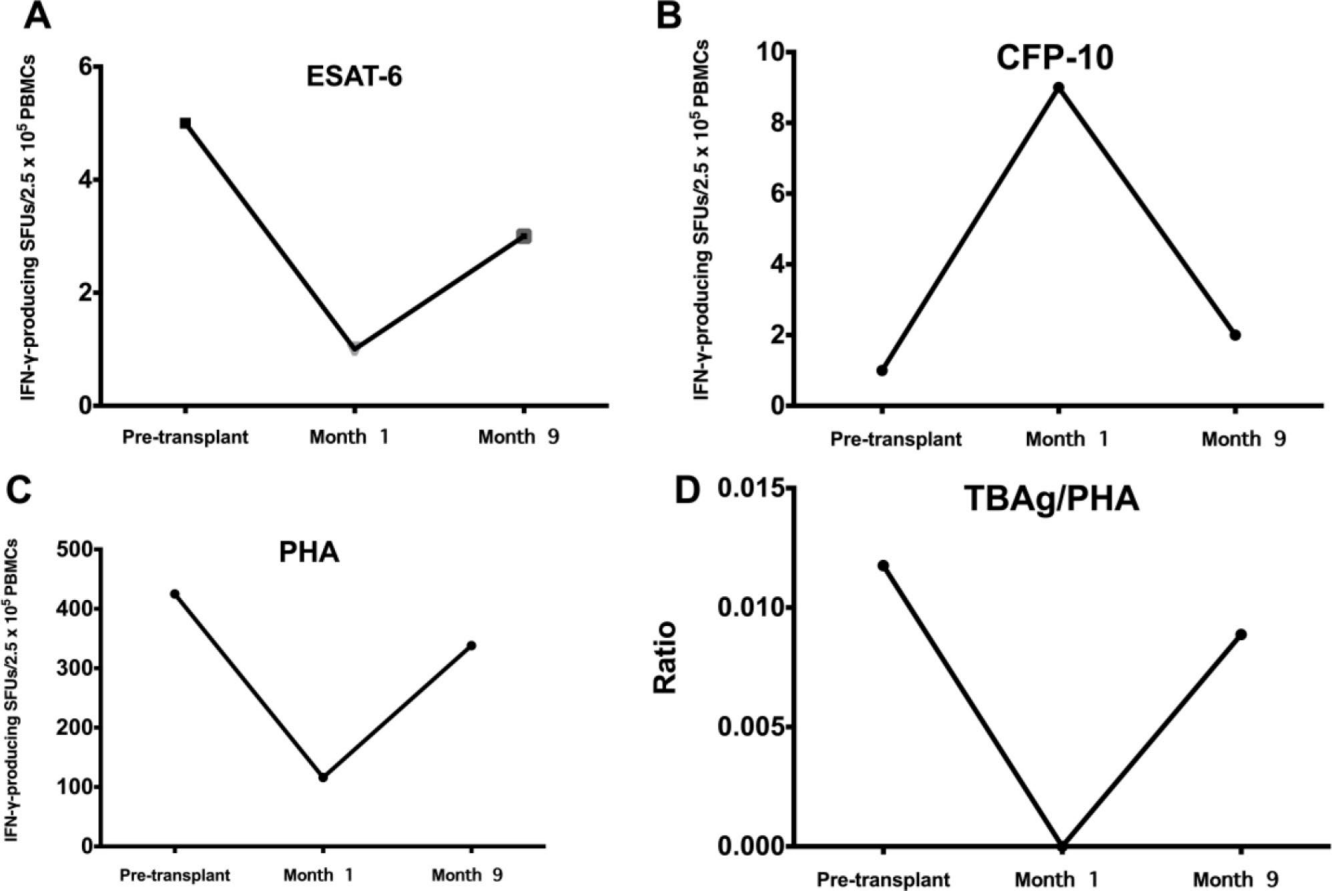
A dynamic plot of TB antigen-specific T cell responses in one kidney transplant recipient who developed pulmonary tuberculosis after transplant. **Abbreviations** CFP-10, culture filtrate protein-10; ESAT-6, early secreted antigenic target 6-kDa protein; PHA, phytohaemagglutinin; PBMCs, peripheral blood mononuclear cells; TBAg, TB antigen; SFU, spot-forming units

Dynamic changes in ESAT-6, CFP-10, and PHA-specific T cell responses, as well as the TBAg/PHA ratio in those with conversion and reversion, are shown in Supplementary Table 1. A dynamic plot of each value in those with positive results and the one who developed TB disease are shown in Figs. 2 and 3, respectively.

## Discussion

We conducted a prospective study to ascertain the incidence of LTBI in KT recipients residing in tuberculosis-endemic areas. Our findings revealed that one-quarter of the participants tested positive for IGRA, indicating potential prior exposure to tuberculosis. Additionally, we observed a higher likelihood of LTBI among KT recipients with advanced age. Notably, our study highlighted a conceivable dynamic alteration in TB-specific T-cell immunity throughout the transplant process.

Thailand is considered an endemic area for TB that is reported to have an incidence of greater than 100 cases per 100,000 population [10]. A plan to screen for LTBI cases and offer an LTBI therapy is essential to reduce the chance of reactivation in order to omit morbidity and mortality from this serious infection. TB is frequently encountered in Thailand, and as such, KT recipients were assumed to carry LTBI status and were universally treated with INH for LTBI without an evaluation of LTBI status. On the other hand, some clinicians would instead monitor clinical presentation of TB to preemptively offer a treatment without LTBI therapy. The incidence of TB in our transplant center where all KT recipients were offered INH therapy regardless of their LTBI status is 0.8%, including severe and disseminated infection. The rate of hepatotoxicity of INH therapy occurred in approximately 16% of the patients, however without severe hepatic failure [11].

In comparison, the other transplant centers in the same setting where a universal INH prophylaxis was not implemented reported an incidence of 4%, almost four times higher [12–14]. However, they were not subjected to medication’s side effects, less pill burden, and drug interactions. We believed a better approach could be performed individually and each specific patient could be managed separately based on their immunologic risk. A pre-transplant LTBI assessment could stratify those at risk and should be offer for LTBI therapy. A study in South Korea revealed a significant proportion of KT recipients with positive IGRA but did not receive INH developed active TB disease greater than those who received the medication [15]. However, a randomized control trial in a high TB burden country like our setting could benefit appropriate management, safety, and cost-effectiveness analysis. A universal INH LTBI therapy could place patients at risk of unnecessary LTBI treatment, drug-induced hepatitis, risk of development of drug resistance if active TB were undetected, risk of drug interactions especially when using rifampicin as an option [14, 16]. A targeted prophylaxis could be more focused and investigated in a well-designed randomized control trial. Furthermore, cost-effectiveness is also important in resource-limited countries. In the meantime, older age is the only factor significantly associated with LTBI in our setting, and this could guide clinicians to be aware of the possibility of LTBI in that particular patient. Older age and history of previous TB have been identified as independent risk factors of LTBI status among Southeast Asian residences (Singapore) [17]. Furthermore, Taiwanese residences with underlying DM are at moderate risk of LTBI [18]. However, self-reported past history of TB diagnosis and underlying DM were not associated with LTBI in our study. Low BMI has been considered as a risk factor for LTBI in previous literature [19]. Disparity of sex as predictive factor for LTBI has been debating even male patients seem to be more predominate [20]. 

About the dynamic change of IGRA, reversion (IGRA positive turn to negative) could be explained by receiving high-dose immunosuppressive drugs that suppressed a cell-mediated immune response. IGRA conversion (IGRA negative turn to positive) typically reflects a new TB disease that found only one patient (1%) in our study (limited by follow-up time). Since there was an early onset of infection with a negative IGRA test prior to KT, a donor-derived infection cannot be entirely excluded. The last reason was false negative at the initial IGRA test. A ratio of ESAT-6 and CFP-10 antigens and phytohemagglutinin ratio has attracted clinicians more to differentiate those with LTBI and active TB infection [21]. At last, we have described those values among KT recipients with positive IGRA test and particularly one who developed TB. However, further studies may need to be explored. We believed LTBI and TB disease could not be distinguished, rather the spectrum of disease is subject to dynamic change dependent on the immune status at any given moment.

One notable strength of this study lies in the successful recruitment of a substantial number of KT recipients. Additionally, the use of the IGRA test enhances sensitivity, particularly beneficial for patients with suppressed T cell function, reducing the risk of false negatives. IGRA also demonstrates superior specificity to *M. tuberculosis*, especially in regions where Bacillus Calmette-Guérin vaccination is prevalent.

Interestingly, we observed that a notable number (one out of five) of those with positive IGRA results prior to transplant turned indeterminate at one month post-transplant, likely attributed to heightened immunosuppression within the initial 30 days post-transplant. This conclusion is supported by the significantly lower PHA response observed during this period compared to prior to transplant. This could raise awareness of false negative results when evaluating with this test after intense immunosuppressant treatment, potentially leading to misinterpretation of TB status in these patients. Additionally, we observed some patients who converted during the course of the transplant but did not develop TB symptoms, likely due to the protective effect of isoniazid prophylaxis. However, one patient who converted did develop clinical TB. He was successfully treated, and the IGRA result eventually reverted. The investigation into dynamic changes in IGRA results during the post-transplant period, an aspect less explored previously, revealed the potential for conversion and reversion influenced by immunosuppressants in this immunocompromised population.

However, the study is not without limitations. A subset of patients was lost to follow-up during concurrent COVID-19 outbreaks, impacting patient care and leading to a lack of blood tests during those visits. Additionally, the focus on the dynamic changes in TB-specific T cell immunity is specific to KT recipients in a setting where universal INH LTBI therapy is employed, within an endemic region. Extrapolating these results to different settings or populations requires further exploration. Additionally, a de novo infection from household contacts cannot be assessed, though the patient was encouraged to report any possible contact with diseases during the study. We also grouped those with borderline results as having a positive result, which could overestimate the true incidence of LTBI. However, we aimed to represent the highest range of data in order to protect these vulnerable patients from disease reactivation.

In conclusion, a noteworthy finding of this study is that one-quarter of KT recipients in a high TB-endemic area tested positive for LTBI using an IGRA. The association between older age and an increased likelihood of carrying LTBI status highlights the importance of personalized TB prevention strategies in KT recipients, tailored to their individual immunological risk. Additionally, clinicians should be vigilant regarding the dynamic changes observed in IGRA responses among KT recipients post-transplant, emphasizing the need for ongoing monitoring and adaptation of preventive measures.

## Electronic supplementary material

Below is the link to the electronic supplementary material.

Supplementary Material 1: Table 1: The dynamic changes of TB antigen-specific T cell responses at each time point in KT recipients with conversion and/or reversion

## Data Availability

The datasets presented in this study are available up on request to the corresponding author.

## Abbreviations

IGRA interferon: gamma release assay
CFP: 10 culture filtrate protein 10
ESAT: 6 early secreted antigenic target 6-kDa protein
KT: Kidney transplant
LTBI: Latent TB infection
PHA: Phytohaemagglutinin
PBMC: Peripheral blood mononuclear cells
TB: Tuberculosis
