# Supplementary material for: Role of interferon-gamma release assay for screening and monitoring of latent tuberculosis infection in kidney transplant recipients
Source: BMC Infect Dis. 2024 Oct 7;24:1110. doi: 10.1186/s12879-024-09990-x (PMC11457419; doi:10.1186/s12879-024-09990-x)
Supplement: Supplementary file 1 — Supplementary Material 1: Table 1: The dynamic changes of TB antigen-specific T cell responses at each time point in KT recipients with conversion and/or reversion [file 12879_2024_9990_MOESM1_ESM.docx]

**Supplementary table 1.** The dynamic changes of TB antigen-specific T cell responses at each time point in KT recipients with conversion and/or reversion.

| **No.** | **IGRA**  **(M0)** | **ESAT-6** | **CFP-10** | **PHA** | **TBAg/PHA ratio** | **IGRA (M1)** | **ESAT-6** | **CFP-10** | **PHA** | **TBAg/PHA ratio** | **IGRA (M9)** | **ESAT-6** | **CFP-10** | **PHA** | **TBAg/PHA ratio** | **Conversion or reversion at month** |
| --- | --- | --- | --- | --- | --- | --- | --- | --- | --- | --- | --- | --- | --- | --- | --- | --- |
|  |  |  |  |  |  |  |  |  |  |  |  |  |  |  |  |  |
| 1 | Positive | 14 | 25 | 308 | 0.08 | Negative | 0 | 0 | 75 | 0.00 | Negative | 0 | 0 | 138 | 0.00 | Reversion M1 |
|  |  |  |  |  |  |  |  |  |  |  |  |  |  |  |  |  |
| 18 | Indeterminate | 21 | 54 | 1006 | 0.05 | Positive | 0 | 11 | 492 | 0.02 | Negative | 0 | 4 | 1012 | 0.00 | Reversion M9 |
|  |  |  |  |  |  |  |  |  |  |  |  |  |  |  |  |  |
| 20 | Indeterminate | 1 | 35 | 1019 | 0.03 | Negative | 2 | 3 | 36 | 0.08 | Positive | 11 | 16 | 620 | 0.03 | ConversionM9 |
|  |  |  |  |  |  |  |  |  |  |  |  |  |  |  |  |  |
| 30 | Negative | 3 | 1 | 474 | 0.01 | Positive | 1 | 5 | 682 | 0.01 | Negative | 0 | 2 | 1333 | 0.00 | Conversion M1 |
|  |  |  |  |  |  |  |  |  |  |  |  |  |  |  |  |  |
| 31 | Positive | 7 | 20 | 939 | 0.02 | Negative | 0 | 0 | 120 | 0.00 | negative | 3 | 3 | 418 | 0.01 | Reversion M1 |
|  |  |  |  |  |  |  |  |  |  |  |  |  |  |  |  |  |
| 40 | Positive | 6 | 3 | 652 | 0.01 | Indeterminate | 0 | 0 | 10 | 0.00 | Negative | 0 | 0 | 1203 | 0.00 | Reversion M9 |
|  |  |  |  |  |  |  |  |  |  |  |  |  |  |  |  |  |
| 45 | Negative | 0 | 3 | 262 | 0.01 | Negative | 0 | 0 | 33 | 0.00 | Positive | 26 | 21 | 1002 | 0.03 | Conversion M9 |
|  |  |  |  |  |  |  |  |  |  |  |  |  |  |  |  |  |
| 53 | Positive | 8 | 5 | 300 | 0.03 | Negative | 0 | 2 | 215 | 0.01 | Negative | 0 | 0 | 995 | 0.00 | Reversion M1 |
|  |  |  |  |  |  |  |  |  |  |  |  |  |  |  |  |  |
| 57 | Negative | 3 | 2 | 55 | 0.05 | Negative | 3 | 1 | 239 | 0.01 | Positive | 1 | 5 | 118 | 0.04 | Conversion M9 |
|  |  |  |  |  |  |  |  |  |  |  |  |  |  |  |  |  |
| 58 | Indeterminate | 0 | 0 | 4 | 0.00 | Negative | 0 | 0 | 152 | 0.00 | Positive | 15 | 11 | 1042 | 0.01 | Conversion M9 |
| 62 | Positive | 10 | 15 | 827 | 0.02 | Negative | 0 | 1 | 358 | 0.00 | Negative | 0 | 0 | 753 | 0.00 | Reversion M1 |
|  |  |  |  |  |  |  |  |  |  |  |  |  |  |  |  |  |
| 64 | Negative | 0 | 3 | 866 | 0.00 | Negative | 0 | 0 | 278 | 0.00 | Positive | 6 | 4 | 1100 | 0.01 | Conversion M9 |
|  |  |  |  |  |  |  |  |  |  |  |  |  |  |  |  |  |
| 65 | Negative | 2 | 2 | 1467 | 0.00 | Positive | 0 | 5 | 138 | 0.04 | Positive | 0 | 6 | 1104 | 0.00 | Conversion M1 |
|  |  |  |  |  |  |  |  |  |  |  |  |  |  |  |  |  |
| 81 | Negative | 0 | 3 | 662 | 0.00 | Indeterminate | 0 | 0 | 0 | 0.00 | Positive | 8 | 0 | 37 | 0.22 | Conversion M9 |
|  |  |  |  |  |  |  |  |  |  |  |  |  |  |  |  |  |
| 85 | Positive | 5 | 1 | 425 | 0.01 | Positive | 1 | 9 | 116 | 0.08 | Negative | 3 | 2 | 338 | 0.01 | Conversion M1 |
|  |  |  |  |  |  |  |  |  |  |  |  |  |  |  |  |  |
| 89 | Positive | 0 | 7 | 159 | 0.04 | Negative | 0 | 4 | 135 | 0.03 | Negative | 0 | 0 | 362 | 0.00 | Reversion M1 |
|  |  |  |  |  |  |  |  |  |  |  |  |  |  |  |  |  |
| 90 | Positive | 7 | 4 | 554 | 0.01 | Negative | 0 | 2 | 54 | 0.04 | Negative | 4 | 0 | 505 | 0.01 | Reversion M1 |
|  |  |  |  |  |  |  |  |  |  |  |  |  |  |  |  |  |

**Abbreviations** CFP-10, culture filtrate protein-10; ESAT-6, early secreted antigenic target 6-kDa protein; M0, pre-transplant; M1, 1 month post-transplant; M9, 9 months post-transplant; PHA, phytohaemagglutinin; PBMCs, peripheral blood mononuclear cells; TBAg, TB antigen; SFU, spot-forming units
